# The state of coursework on horses in human services at universities and colleges in the United States: a scoping review

**DOI:** 10.3389/fvets.2023.1305353

**Published:** 2023-11-28

**Authors:** Katherine Connolly, Nina Ekholm Fry

**Affiliations:** Graduate School of Social Work, Institute for Human-Animal Connection, University of Denver, Denver, CO, United States

**Keywords:** horse, equine, equine-assisted therapy, equine-assisted services, college, university, higher education, course

## Abstract

An increasing number of universities and colleges in the United States are offering coursework on adaptive/therapeutic riding and the incorporation of horses in human service areas such as psychotherapy, education, occupational therapy, physical therapy, and speech-language pathology. The first study to identify coursework in these areas was published in 2018. In order to track development over time, we conducted a replication study to determine the prevalence of coursework on horses in human services at higher education institutions. Information gathered for the 2021–2022 academic year included the institution name, geographic location, number of courses and their focus, academic department offering the course, and level of study. We identified 122 courses provided by 48 higher education institutions in 29 states in the following areas: adaptive/therapeutic riding (*N* = 82, 67.2%), mental health (*N* = 19, 15.6%), education/learning (*N* = 2, 1.6%), and equine movement in physical therapy, occupational therapy, and speech-language pathology (hippotherapy) (*N* = 1, 0.8%). Survey or overview courses (*N* = 18, 14.8%) were also identified. These courses were offered both at the undergraduate (*N* = 114, 93.4%) and the graduate level (*N* = 8, 6.6%) by a total of 48 departments that either focused on animals, such as equine science, animal science, and agriculture (*N* = 27, 54%) or focused on humans, such as health science or liberal arts (*N* = 23, 46%). The results inform a discussion on changes over time as well as current challenges and opportunities for academic programs offering coursework about horses in human services.

## 1 Introduction

Interest in the inclusion of horses in human services and in adaptive/therapeutic riding continues to grow in the United States of America (USA). There are several human service and activity areas where horses are involved, which determine the training required of the professional providing the service. In the U.S., horses are included in two broad areas of human services: in therapy services and in education or learning services (1). Therapy services involve a licensed therapist with additional training who may incorporate elements of equine interactions, equine movement, and the equine environment in their overall treatment approach and plan, tailored to the client’s needs and goals (2). Examples include the use of equine movement (hippotherapy) in physical therapy, occupational therapy, or speech-language pathology services (3) and interactions with horses that are incorporated into psychotherapy (4). Professionals providing educational or learning services that are based on learning frameworks and focused on goals related to areas such as skill development, academic achievement, or organizational development can include equine interactions to enhance the service they are providing (1). Adaptive riding, a term used concurrently with therapeutic riding and therapeutic horsemanship lessons (stylized in this publication as adaptive/therapeutic riding) is provided by an instructor and provides access to horses and horsemanship activities for those who may experience disability-related barriers for interacting with horses in typical equestrian environments or who otherwise need accommodations when doing so (1).

Coursework focused on horses in human services and in adaptive/therapeutic riding at universities and colleges in the United States has become more common. Undergraduate and graduate students who are interested in pursuing a career that includes interacting with horses within these services or activities can find a variety of programs, courses, majors, minors, and certificates offered by higher education institutions. The content of these offerings varies widely in prerequisites, scope, specificity, learning objectives, and cost (5). With the exception of a conference that was organized by Middle Tennessee State University in 2014, specifically addressing coursework of this nature in higher education (6), no dedicated network exists for academic professionals who administer coursework in these areas. This may contribute to delays in adopting the latest terminology, concepts, and guidelines, and a lack of clarity about what constitutes foundational and advanced knowledge in the focus area of the coursework. The Professional Association of Therapeutic Horsemanship International (PATH Intl.) offers a higher education membership to academic institutions whose coursework enables students to become certified primarily in adaptive/therapeutic riding or that offer activities in this area at their college equestrian center (7). At the time of publication, 12 higher education institutions are members.

Coursework on horses in human services and adaptive/therapeutic riding in higher education has not received much attention in previous literature. A total of eight English-language articles were identified (5, 8–14), of which four were published as conference abstracts (8, 9, 13, 14). These articles focused primarily on adaptive/therapeutic riding, including feasibility to implement coursework (12) and the education and skills preferred by adaptive/therapeutic riding centers when hiring staff (10). In addition, four doctoral dissertations related to higher education coursework were found (15–18). Service-based learning opportunities in undergraduate programs involving adaptive/therapeutic riding have also been discussed (11, 19, 20).

With the exception of a survey focusing on the number of academic institutions with connections to North American Riding for the Handicapped Association (NARHA) adaptive/therapeutic riding centers (NARHA is now PATH Intl.) (8), the first comprehensive review of coursework in U.S. universities and colleges concerning horses in human services and adaptive/therapeutic riding was published in 2018 (5). The study, which was led by the second author of the present study, found 110 courses offered in the 2016–2017 academic year at 39 U.S. higher education institutions (universities and colleges). Courses were categorized per course content focus area, per geographic location (state), per level of learning (undergraduate or graduate), and per the department or academic area where the course was offered.

The purpose of the present study was to determine the current prevalence and nature of coursework focused on horses in human services and adaptive/therapeutic riding in U.S. higher education institutions through a comprehensive scoping review, which included number of courses, number of higher education institutions, geographic location, course focus area, level of learning, and department or academic area for the 2021–2022 academic year. In order to track development in this area over time, we replicated the most recent review conducted in this area (5).

## 2 Methods

As no registry or database exists for coursework involving horses in human services and adaptive/therapeutic riding, and in order to replicate the 2018 study (5), we conducted a scoping review via internet search. Scoping reviews allow researchers to identify and map the available evidence and identify knowledge gaps, and are especially appropriate when a topic has minimal preexisting academic research (21). We consulted the preferred reporting items for systematic reviews and meta-analyses extension for scoping reviews (PRISMA-ScR) in our reporting (22).

### 2.1 Inclusion criteria

We used the following criteria for selecting courses for inclusion in this review:

- The course was offered by an accredited post-secondary academic institution (university or college) in the United States.
- The course was listed in the 2021–2022 publicly accessible, online academic course catalog, or, in the case of academic certificates, an online record of certificate being offered during 2021 or 2022.
- The course title and/or description referenced adaptive riding/therapeutic riding or specified the incorporation of horses or equines in mental health; physical therapy, occupational therapy, speech-language pathology; education/learning, or was described as a survey course on one or several of these areas.

Special topics courses, elective courses, internships, and practicums that were not clearly identified in the course catalog as meeting criteria (c) were excluded from the review.

### 2.2 Information sources and search procedure

The search was conducted between April, 2022 and January 2023 (10 months) using the internet search engine Google. The search terms used were “equine,” “horse,” “therapeutic,” “adaptive,” “equine-assisted,” “facilitated,” and “human-horse,” paired with “course,” “university,” “college,” and “higher education.” Examples of search results included a course or program webpage, a course catalog entry, a news story or other informational item related to the institution. After evidence of a course and accompanying academic institution was identified through search results, we located the 2021–2022 course catalog through the institution’s website search function and searched it using the following terms: “equine,” “horse,” “therapeutic,” “adaptive,” “equine-assisted,” “facilitated,” “therapy,” “learning,” and “human-horse.” In addition, when a course that met all three inclusion criteria was identified, it was cross-checked with the dataset used in the 2018 study.

### 2.3 Data extraction and categorization

During data extraction, we recorded the following information: the course title and description, the name of the institution offering the course, the geographic location of the institution, the level of study (undergraduate or graduate), and the academic department or academic area through which the course was offered. All items could be accessed for each course.

In the United States, undergraduate courses are offered within four-year bachelor’s degree programs, and two-year associate degree programs. In this study, we defined graduate-level courses as those offered within master or doctoral programs, post-masters programs, or other graduate-level certificate programs offered by a university. Following the groupings created in the 2018 study (5), we categorized departments or academic areas as either primarily focused on animals, such as equine science, animal science, and agriculture, or on humans, such as health science, social science, and liberal arts.

### 2.4 Determination of course content focus area

To track development over time in coursework prevalence, we used the same five categories created in the 2018 study (5) to determine course focus area. Due to updates in terminology and concepts, we renamed and updated the descriptions for each category (1). This did not substantially alter the nature of the categories. Importantly, when determining the focus area of each course, it was necessary to evaluate the nature of course content not simply through terms used but through the context provided by the course description. All focus area determinations were rated by both authors and any discrepancies were resolved by discussion. We used the following descriptions for the focus areas.

#### 2.4.1 Adaptive/therapeutic riding

Adaptive riding, also known as therapeutic riding and therapeutic horsemanship (1), is the provision of riding and other horsemanship lessons to individuals who may experience disability-related barriers for interacting with horses in typical equestrian environments or who otherwise need accommodations when doing so. This is provided by a riding instructor.

#### 2.4.2 Mental health

Mental health professionals, such as clinical psychologists, mental health clinical counselors, clinical social workers, marriage and family therapists, psychiatrists, and psychiatric nurse practitioners who provide psychotherapy or clinical counseling, may include equine interactions within their theoretical orientation and chosen therapy approaches with the purpose of enhancing treatment outcomes for the client if specifically trained to do so (4).

#### 2.4.3 Education/learning

Educational or learning-based services are based on learning frameworks and focused on goals related to areas such as skill development, academic achievement, and organizational development. Professionals working in these areas can include equine interactions to enhance the service they are providing (1).

#### 2.4.4 Equine movement in physical therapy, occupational therapy, and speech-language pathology

Occupational therapists, physical therapists, and speech-language pathologists use purposeful manipulation of equine movement as a therapy tool, also known as hippotherapy, to engage sensory, neuromotor and cognitive systems to promote functional outcomes within the patient’s plan of care (2).

#### 2.4.5 Survey/overview course

This category comprised of survey, overview, and introductory courses that included an overview of several focus areas.

### 2.5 Comparison with previous scoping review

We compared the results of our scoping review with the results of the 2018 study (5) to track changes over time.

## 3 Results

### 3.1 Courses and institutions

A total 122 courses were offered at 48 higher education institutions across the United States.

### 3.2 Geographic location

**Figure 1:**
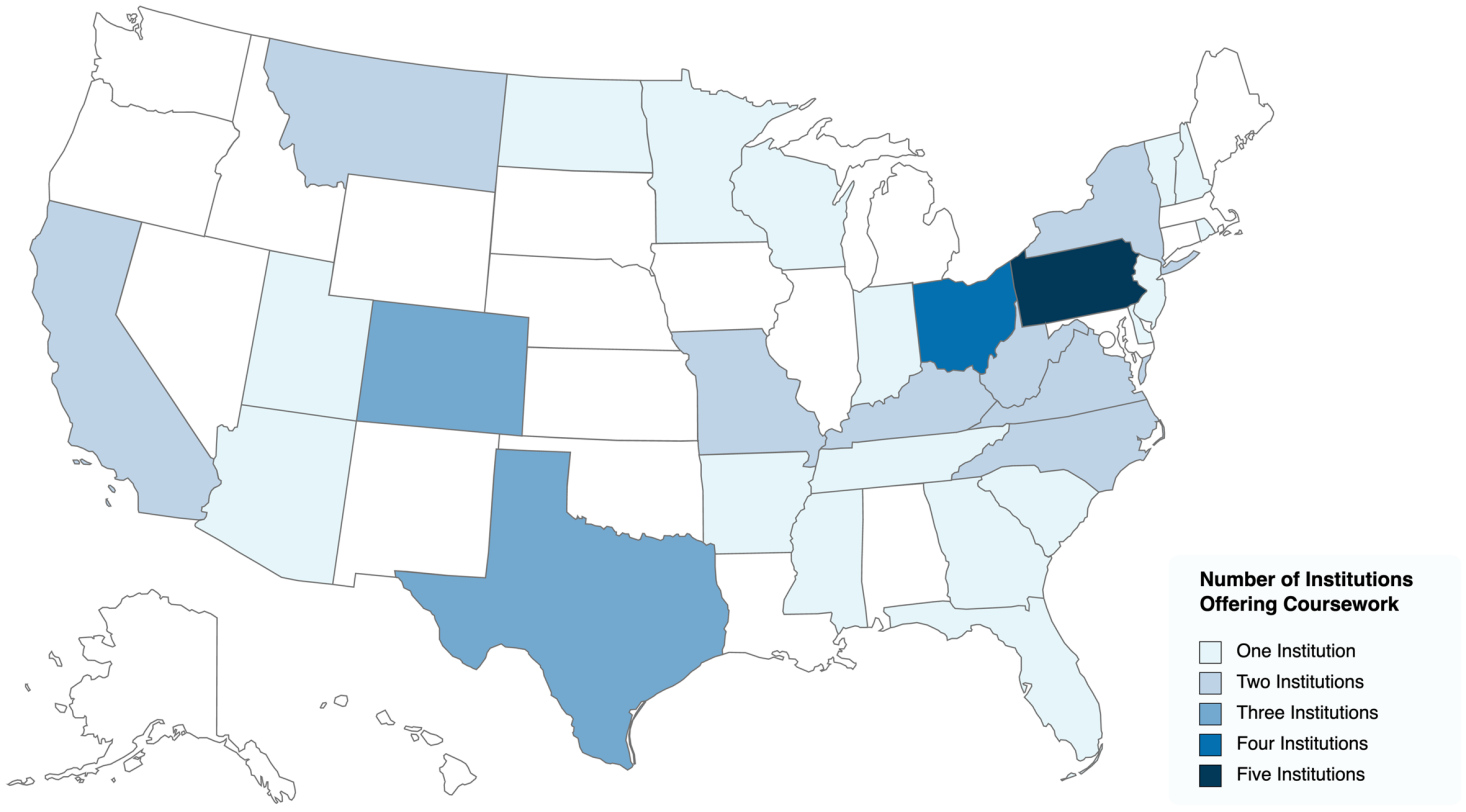
A map of the number of higher education institutions per state offering coursework on horses in human services and adaptive/therapeutic riding in the 2021–2022 academic year.

Higher education institutions in a total of 29 states (57.9%) offered coursework while 21 states did not have any academic institutions offering relevant coursework (Figure 1). The state with the largest number of courses offered was North Carolina (*N* = 14) and the state with the largest number of higher education institutions offerings coursework was Pennsylvania (*N* = 5). Except for the Pacific Northwest, all major geographic areas of the contiguous United States had higher education institutions offering relevant coursework. No coursework was identified in Hawaii or Alaska.

### 3.3 Course content focus area

Categorizing the 122 courses according to content focus resulted in the following: adaptive/therapeutic riding (*N* = 82, 67.2%), mental health (*N* = 19, 15.6%), education/learning (*N* = 2, 1.6%), equine movement in physical therapy, occupational therapy, and speech-language pathology (*N* = 1, 0.8%), and overview/other (*N* = 18, 14.8%) (see Table 1). The largest number of courses was offered in the area of adaptive/therapeutic riding (*N* = 82) and the smallest number of courses was on the topic of equine movement in physical therapy, occupational therapy, and speech-language pathology (*N* = 1).

**Table 1:** Coursework by focus area on horses in human services and adaptive/therapeutic riding in the 2021–2022 academic year compared with the 2016–2017 academic year, as reported by Ekholm Fry et al. (5).

	Total courses	Adaptive/Therapeutic riding	Mental health	Education/Learning	Equine movement in PT, OT, or SLP^*^	Survey/Overview
2021–2022	122	82	19	2	1	18
2016–2017^†^	110	71	23	7	1	8

^*^PT, Physical therapy; OT, occupational therapy; SLP, speech-language pathology. ^†^Data from Ekholm Fry et al. (5).

### 3.4 Level of learning

A total of 114 courses (93.4%) at 43 institutions were offered at the undergraduate level and a total of 8 courses (6.6%) at 5 institutions were offered at the graduate level. If a course was open to both undergraduates and graduate students, it was counted only once and categorized as an undergraduate course.

### 3.5 Department

A total of 48 departments or academic areas provided relevant coursework at 48 higher education institutions. Of the 48 departments offering coursework, 26 (54.2%) were focused on animals, such as equine science, animal science, and agriculture and 22 (45.8%) were focused on humans, such as health science, social science, and liberal arts. From a course perspective, a total of 70 courses (57.4%) were offered by primarily animal-focused departments and 52 (42.6%) by primarily human-focused departments.

### 3.6 Comparison to previous scoping review

When comparing results from the present study for academic year 2021–2022 with results from the academic year 2016–2017 (5), we found that the number of total courses offered has increased by 12, from 110 to 122 courses total. The number of higher education institutions offering courses has increased by 9, from 39 to 48 institutions. Geographically, the same number of states have institutions offering relevant coursework (*N* = 29). There was an increase in undergraduate courses, from 93 in 2016–2017 to 114 in 2021–2022, totaling 21 courses. Meanwhile, there was a decrease in graduate courses, from 17 in 2016–2017 to 8 in 2021–2022, totaling 9 courses. The number of departments offering relevant coursework has increased by 11, from 39 to 48. The percentage of coursework offered either through primarily animal-focused or human-focused departments has changed slightly. In the academic year of 2016–2017, coursework was offered by 19 departments (48.7%) that were focused on animals, such as equine science, animal science, and agriculture, and by 20 (51.3%) departments focused on humans, such as health science, social science, or liberal arts. In the academic year of 2021–2022, coursework was offered by 27 departments (54.0%) focused on animals, and by 23 (46.0%) focused on humans (see Table 2). Regarding course focus area, there was an increase in adaptive/therapeutic riding courses and survey/overview courses, and a decrease in mental health and education/learning courses. There was no change in courses offered on equine movement in physical therapy, occupational therapy, and speech-language pathology (see Table 1).

**Table 2:** Coursework on horses in human services and adaptive/therapeutic riding in the 2021–2022 academic year compared with the 2016–2017 academic year, as reported by Ekholm Fry et al. (5).

	Total courses	Total institutions	Total states	Total undergraduate courses	Total graduate courses	Total departments	Total departments per type
2021–2022	122	48	29	114	8	48	27 (a)^*^ 23 (h)^**^
2016–2017^†^	110	39	29	93	17	39	19 (a)^*^ 20 (h)^**^

^*^Animal-focused departments (e.g., equine science, animal science, agriculture). ^**^Human-focused departments (e.g., health science, social science, liberal arts). ^†^Data from Ekholm Fry et al. (5).

## 4 Discussion

We conducted a study focused on the prevalence of coursework in U.S. higher education in areas of horses in human services and adaptive/therapeutic riding for the academic year of 2021–2022. This study successfully replicated the first comprehensive scoping review in this area (5) and provides data for tracking coursework development over time. Our results show an increase in coursework offered by higher education institutions by comparing offerings in the academic year of 2016–2017 with 2021–2022. By recording the number of courses and institutions, the geographic location of the institution, the focus of course content, the level of study (undergraduate or graduate), and the academic department or academic area through which the course was offered, we discovered several notable developments.

An additional 9 academic institutions started offering coursework in the five-year time period covered by the data comparison, bringing the total number of institutions from 39 in 2016–2017 to 48 in 2021–2022. In addition, 12 additional courses were offered in 2021–2022, primarily at the undergraduate level. The number of departments offering coursework increased from 39 to 48. Institutions offering relevant coursework are located in over half of U.S. states and location by state remained more or less the same in the comparison period. When considering course focus, the largest increases were found in the number of adaptive/therapeutic riding courses and in the survey/overview courses offered. One exception to the overall increase in coursework was in the focus areas of horses in mental health and horses in education where a decrease in offerings was found. One institution that in 2016–2017 had offered 10 courses total at the graduate level, five courses in mental health and five in education/learning, had stopped doing so by 2021–2022, which likely has a large impact in those areas. This may have also impacted the overall number of courses offered at the graduate level, which had declined by 2021–2023. The percentage of courses offered primarily through departments focused on animals, such as equine science, animal science, and agriculture, in contrast with those focused on human health science, social science, or liberal arts had also changed somewhat between the two comparison points. In the 2016–2017 academic year, 48.7% of coursework was offered by departments focused on animals, and 51.3% by departments focused on humans. In the 2021–2023 academic year, 56.6% of coursework offered by departments focused on animals and 43.4% by departments focused on humans.

While coursework in the relevant areas has increased, the issues noted in the previous scoping review have remained, namely those surrounding terminology and conceptualization of services and activities that involve horses. These challenges have been discussed extensively in recent years (1, 4). Issues concerning the terms used to describe services and how to understand interactions with horses in human services and adaptive/therapeutic riding impacted the current study in several ways. Identifying and categorizing coursework is dependent on shared understanding of what is described. The most challenging aspect of conducting this scoping review was to discern what a course was about based on the available course title and description. Often, the course title included the word “therapy,” but the course description focused solely on adaptive/therapeutic riding, referencing riding lessons, instructions, adapted equipment, and such. Categorizing such a course offering in one of the two healthcare service areas, mental health or equine movement in occupational therapy, physical therapy, or speech language pathology, would have been incorrect, although these are the only areas that involve therapy. As such, conducting any kind of review in the area of horses in human services and in adaptive/therapeutic riding requires considerable knowledge about the nature of services and activities, not just commonly used terminology, as this may be used inappropriately. Using combinations of words that involve “therapy” or “intervention” while meaning adaptive/therapeutic riding emerged as the largest issue concerning terminology for higher education institutions to address, as the majority of course offerings are focused on adaptive riding lessons for individuals with disabilities.

Appropriate conceptualization of horses in human therapy and learning services and the separate activity of adaptive/therapeutic riding is also necessary in order to not mislead students about course offerings. For instance, a total of 13 courses with a specific focus on therapy services were offered at the undergraduate level in 2021–2023. When courses that focus on therapy services where equine interactions are included are offered at the undergraduate level, it should be clear to students that they cannot provide psychotherapy, occupational therapy, physical therapy, or speech language therapy independently without a master’s degree in the U.S. and that this does not change when equine interactions are included as part of the service. The choice of department by which a course about horses in human services is offered is another interesting aspect. Unlike education/learning services, where no specific level of academic training is required for professionals in most cases, human services, such as psychotherapy, require advanced academic degrees and licensure in order for the professional to provide the service. As such, courses offered by animal-focused departments may be limited in how they are able to prepare students for professional work in human services. In light of these limitations, it makes sense that adaptive/therapeutic riding, the largest area of coursework by far, is offered at an undergraduate level by primarily animal-focused departments. Interestingly, in a recent survey of adaptive/therapeutic riding center staff involved in hiring decisions, less than half (42%) indicated that having a bachelor’s degree was a major consideration in hiring for a management position, and only 22% indicated that they “strongly agreed” or “agreed” that having a bachelor’s degree with major/minor in adaptive/therapeutic riding or with a focus on horses in human services was important (10).

Another conceptual challenge arises when institutions offer minors within bachelor’s degrees with titles that reference horses in human health or adaptive/therapeutic riding, but do not offer any courses specific to the topic area. Instead, students may complete credits in areas such as human development, anatomy, equine science, and equine business. Institutions with minors or tracks built this way were not included in this study as they did not meet the inclusion criteria. It is arguably misleading for students to pursue a minor where they do not get direct instruction in the topic area they are interested in.

The delineation of and descriptions used for the four main course focus areas in this study follow currently proposed terminology and conceptual guidelines (1, 2, 4). As the most current national guidelines were published immediately preceding the 2021–2022 academic year, it is not expected that coursework included in this review would be informed by them. Perhaps due to the lack of opportunities for professional exchange between instructors who offer academic courses in these unique areas, there appears to be large differences in course content, which only further contributes to issues with terminology and conceptualization. Based on course descriptions reviewed in this study, no agreement seems to exist regarding what constitutes basic or introductory knowledge in these distinct course topic areas, and, as a result, what should be included in survey courses or taught at different academic levels.

### 4.1 Limitations and future directions

As no database exists outside of course catalogs for individual institutions, it is possible that courses meeting criteria have been omitted due to not having been found during internet searches. In addition, the inclusion criteria leave out student-designed electives and other academic activities taking place outside of standard courses. Course offerings are not static, and the data provided in this article is specific to what took place during the 2021–2022 academic year. The possible impact of COVID on the development of new course offerings and delivery of existing courses in this area should also be taken into consideration when reviewing the data.

Our suggestions for future research echo those proposed in the 2018 review (5) and feel particularly critical in light of the increase in course offerings. Examination of curricula and their connection to competencies and standards of professional practice in the various areas of coursework is of central importance. Surveying instructors and course creators could result in telling information about how course content varies across institutions. Finally, an increase in support and information exchange between instructors and academic units across institutions may serve to strengthen the development and quality of course offerings in this area.

## Data availability statement

The datasets presented in this article are not readily available because identifiable data for institutions will be withheld. Requests to access the datasets should be directed to nina.ekholm-fry@du.edu.

## Author contributions

KC: Conceptualization, Data curation, Methodology, Visualization, Writing – original draft, Writing – review & editing. NEF: Conceptualization, Methodology, Supervision, Writing – original draft, Writing – review & editing.

## References

[1] Wood W Alm K Benjamin J Thomas L Anderson D Pohl L . Optimal terminology for services in the United States that incorporate horses to benefit people: a consensus document. J Altern Complement Med. (2021) 27:88–95. doi: 10.1089/acm.2020.0415, PMID: 33252244

[2] American Hippotherapy Association. AHA, Inc. terminology for healthcare. (2020). Available at: http://www.americanhippotherapyassociation.org/resources-2/

[3] American Hippotherapy Association. Statements of best practice for the use of hippotherapy by occupational therapy, physical therapy, and speech-language pathology professionals. (2021). Available at: http://www.americanhippotherapyassociat ion.org/resources-2/

[4] Ekholm FN. Conceptualization of psychotherapy incorporating equine interactions in the United States. Hum Anim Interact Bull. (2021) 9:94–114. doi: 10.1079/hai.2021.0036

[5] Ekholm Fry N Meszaros E O’Neill K. Coursework in equine-assisted activities and therapies at universities and colleges in the United States: a scoping review. Hum Anim Interact Bull. (2018) 6:118–26. doi: 10.1079/hai.2018.0021

[6] Middle Tennessee State University. MTSU news: MTSU takes reins in hosting therapeutic horse conferences. (2014). Available at: https://mtsunews.com/mtsu-hosts-horse-conferences/

[7] PATH Intl. higher education membership program. Available at: https://pathintl.org/membership/higher-ed/ (Accessed May 30, 2023).

[8] Brady HA Hernandez HM Guay KA. The status of equine-assisted therapy programs within higher education. J Equine Vet. (2011) 31:350. doi: 10.1016/j.jevs.2011.03.194

[9] Burk S Gramlich C. 163 college curricula valued by equine-assisted activity and therapy centers. J Equine Vet. (2015) 35:453. doi: 10.1016/j.jevs.2015.03.176

[10] Burk S Gramlich C. College coursework and skills valued for employment in the equine-assisted activities and therapies field 1. NACTA J. (2018) 62:16–22.

[11] Brady HA Lawver DE Guay KA Pyle AA Cepica NT. Principles of therapeutic riding as a service based learning course within an agricultural curriculum. NACTA J. (2005) 49:19–23.

[12] Colston C Shultz A Porr C. The feasibility of implementing an equine-assisted activities and therapy curriculum into higher education 1. NACTA J. (2015) 59:189–91.

[13] Murphy BA. Development of equine assisted therapy and learning as an elective module within an Irish equine curriculum. J Equine Vet. (2011) 31:350–1. doi: 10.1016/j.jevs.2011.03.195

[14] Nicodemus M. Student confidence levels in horsemanship skills associated with a university equine assisted therapy course. J Equine Vet. (2011) *31*:332. doi: 10.1016/j.jevs.2011.03.171

[15] Colston C. The feasibility of implementing equine-assisted activities and therapy curriculum in higher education [dissertation]. Murray, KY: Murray State University (2014).

[16] Mullen G. Integrating equine-assisted activities and therapy (EAAT) into a higher learning institution [dissertation]. Minneapolis, MN: Walden University (2010).

[17] Rothman N. Exploring the professional development of MFT students enrolled in equine assisted family therapy coursework: an experiential learning modality [dissertation]. Davie, FL: Nova Southeastern University (2020).

[18] Shkedi A. Equine assisted activities or therapy: towards a future curriculum [dissertation]. England: University of Derby (2015).

[19] Pyle AA Brady HA Lawver DE Akers CL Cepica NT. Journal-based reflection in undergraduate service learning and the university therapeutic riding center. NACTA J. (2004) 48:72–3.

[20] Vogelsang MM Lazo M Harper K Shehane M. Equine assisted and affiliated therapies provide service-learning opportunities for students. J Anim Sci. (2016) 94:64. doi: 10.2527/ssasas2015-132

[21] Munn Z Peters MDJ Stern C Tufanaru C McArthur A Aromataris E. Systematic review or scoping review? Guidance for authors when choosing between a systematic or scoping review approach. BMC Med Res Methodol. (2018) 18:143. doi: 10.1186/s12874-018-0611-x, PMID: 30453902 PMC6245623

[22] Tricco AC Lillie E Zarin W O'Brien KK Colquhoun H Levac D . PRISMA extension for scoping reviews (PRISMA-ScR): checklist and explanation. Ann Intern Med. (2018) 169:467–73. doi: 10.7326/M18-0850, PMID: 30178033

